# Relationship of C-peptide levels to duration of Type 1 diabetes – A study from Sindh, Pakistan

**DOI:** 10.12669/pjms.36.4.1531

**Published:** 2020

**Authors:** Asher Fawwad, Nazish Waris, Saima Askari, Graham Ogle, Muhammad Yakoob Ahmedani, Abdul Basit

**Affiliations:** 1 Asher Fawwad, PhD. Professor & Head of the Biochemistry Department (BMU), Honorary Research Director (BIDE), Baqai Institute of Diabetology and Endocrinology (BIDE), Karachi, Pakistan. Baqai Medical University (BMU), Karachi, Pakistan.; 2 Nazish Waris, M.Phil. Clinical Biochemistry and Psychopharmacology Research Unit Department of Biochemistry, University of Karachi-Pakistan Research Associate, Research Department (BIDE-BMU), Baqai Institute of Diabetology and Endocrinology (BIDE), Karachi, Pakistan. Baqai Medical University (BMU), Karachi, Pakistan.; 3 Saima Askari, Fellow Endocrine, (BIDE-BMU), Baqai Institute of Diabetology and Endocrinology (BIDE), Karachi, Pakistan. Baqai Medical University (BMU), Karachi, Pakistan.; 4 Graham David Ogle, General Manager, Life for a Child Program, Diabetes NSW, Glebe, NSW, Sydney, Australia; 5 Muhammad Yakoob Ahmedani, FCPS, Professor of Medicine, Department of Medicine (BIDE-BMU), Baqai Institute of Diabetology and Endocrinology (BIDE), Karachi, Pakistan. Baqai Medical University (BMU), Karachi, Pakistan.; 6 Abdul Basit, FRCP, Professor of Medicine (BMU), Director (BIDE), Baqai Institute of Diabetology and Endocrinology (BIDE), Karachi, Pakistan. Baqai Medical University (BMU), Karachi, Pakistan.

**Keywords:** Type 1 diabetes, C-peptide, Duration of diabetes

## Abstract

**Objective:**

To determine the relationship of C-peptide levels with duration of type 1 diabetes mellitus.

**Methods:**

This prospective study was conducted at Baqai Institute of Diabetology and Endocrinology (BIDE), Baqai Medical University (BMU), Karachi-Pakistan from December 2013 to December 2015. A total of 184 subjects were recruited during the study period, 100 in Group-A and 84 in Group-B. Subjects clinically diagnosed with type 1 diabetes Mellitus (T1DM) were categorized into two groups based on duration of diabetes: Group-A (with ≤1-year duration of diabetes) and Group-B (with >1-year duration of diabetes). Ninety-nine of the 100 enrolled subjects in Group-A were diagnosed as having T1DM, with one subject who presented at 11.9 years of age and diagnosed with T2DM excluded from this study. Blood samples were drawn for biochemical parameters. Data for baseline characteristics and clinical parameters (HbA1c and C-peptide) were obtained from hospital management system of BIDE.

**Results:**

Fifty-seven (57.6%) subjects in Group-A, and 39 (46.4%) in Group-B were males. Mean±SD duration of diabetes (years) was 0.64±0.6 (range 0-1) in Group-A, and 7.65±5.5 (range 1-23) in Group-B. Family history of T1DM and T2DM was 1(1%) and 27(27.3%) in Group-A, and 8(9.52%) and 21(25%) in Group-B, respectively. Twenty-one (21.2%) subjects presented in diabetic ketoacidosis (DKA) in Group-A and 18(21.4%), in Group-B. Mean±SD for HbA1c was non-significantly higher in Group-A 11.12±2.31 compared to Group-B 10.42±1.45. Mean±SD for C-peptide was 1.91±1.53 ng/mL (0.60±0.481 nmol/L) in Group-A, and 1.82±1.01 (0.57±0.32 nmol/L) in Group-B (p=0.984).

**Conclusion:**

The study found that subjects with longer duration of T1DM had non-significantly decreased C-peptide levels compared to a group in which C-peptide was measured at or soon after diagnosis. Furthermore, C-peptide levels in many subjects with longer duration were higher than expected in classic T1DM.

## INTRODUCTION

The hallmark of type 1 diabetes mellitus (T1DM) is absence or very low levels of insulin production, which can be indirectly seen by the presence of low C-peptide levels.^1^,^2^ Approximately 40-50% of the risk of disease arises from genetics, while the remaining risk arising from poorly defined environmental etiologies.^3^ T1DM can be subdivided in various ways. One subdivision identifies Type 1A, resulting from autoimmune mediated destruction of β-cells, and Type 1B which is less prevalent and mostly seen in Asian and African population with unknown etiology, which present with variable degrees of insulin deficit and residual β-cell function.^4^,^5^

Pancreatic islet-cells of Langerhans secrete insulin and C-peptide in equimolar amounts into the blood in response to hyperglycemia. C-peptide is mainly excreted by the kidney with a half-life three-four times longer than that of insulin. It is used to assess endogenous insulin secretion in patients with T1DM.^6^ In most of the patients with long-duration of T1DM, very low levels of C-peptide can still be detected. The prevalence of detectable C-peptide in patients with T1DM depends on several factors including duration of diabetes, age at diagnosis, and sensitivity of assays used.^7^

The physiological action and potential protective role of preservation of C-peptide secretion in T1DM has been keenly investigated in the last few years.^8^ Various studies, including the Diabetes Control and Complications Trial and experimental studies, have described the beneficial role of C-peptide in diabetic complications in animal models and T1DM patients as studies suggest that preservation of C-peptide secretion helps to prevent or delay diabetes-related vasculopathy.^9^,^10^ Thus, the aim of this study was to determine the relationship of duration of clinically-diagnosed T1DM with C-peptide levels in young Pakistanis.

## METHODS

This prospective study was conducted at Baqai Institute of Diabetology and Endocrinology (BIDE), Baqai Medical University (BMU), a tertiary care unit in Karachi-Pakistan. The duration of study was between December 30, 2013 to December 23, 2015, and was an additional investigation from the main study “Epidemiology of youth-onset T1DM in Pakistan: clinical features, biochemistry and HLA-DRB1”.^11^ Ethical approval was obtained from the Institutional Review Board (IRB) of BIDE with Ref no: BIDE/IRB/Prof.Yakoob/03/27/13/119. Written informed consent was obtained for subjects >18 years prior to enrolment in the study, and for subjects <18 years, their parents/guardians gave written informed consent. Data was obtained from the hospital management system (HMS) of BIDE.

Diabetes was clinically diagnosed according to standard World Health Organization (WHO) criteria.^12^ Determination of the type of diabetes was made by local investigators according to available clinical features and history. Subjects were categorized into two groups based on duration of T1DM. Group-A consisted of subjects with ≤1-year duration of diabetes, and Group-B has >1-year duration of diabetes. Subjects without diabetes or with diagnosis other than T1DM were excluded from this study. Blood samples were drawn for clinical parameters. Data for baseline characteristics including age, gender, duration of diabetes, family history of T1DM and T2DM (father, mother and siblings), BMI and clinical parameters (HbA1c and C-peptide) were obtained from hospital management system of BIDE. The presence of Diabetic ketoacidosis (DKA) was recorded defined as the presence of ketonuria, as well as acidosis (anion gap calculation and/or measurement of arterial blood gases).

Peripheral blood was collected by venepuncture into vacutainer tubes on the day of assessment, after an overnight fast. Serum samples were spun down immediately and stored in a -20 °C freezer. HbA1c (%) was measured by high-performance liquid chromatography method. Fasting C-peptide was measured from frozen samples by commercially available ELISA kits (IBL, Hamburg, Germany) in Pakistan. Reference range of C-peptide was considered as 0.8-3.1 ng/mL (0.26-1.03 nmol/L).^13^,^14^ C-peptide was further categorized as <0.4 ng/mL (<0.13 nmol/L), 0.4-0.8 ng/mL (0.13-0.26 nmol/L) and >0.8 ng/mL (>0.26 nmol/L).

All data were computed and analyzed using Statistical Package for Social Sciences (SPSS), (version 20.0). Continuous data were expressed as the mean±standard deviation (SD) and categorical variables were presented as n (%). Students t-test, Mann-Whitney U test, Fisher exact test or Chi-squared test were applied where applicable.

## RESULTS

A total of 184 subjects were recruited during the study period, 100 in Group-A and 84 in Group-B. Group-A were a consecutive series of diagnoses. Ninety-nine of the 100 enrolled subjects in Group-A were diagnosed as having T1DM, with one subject who presented at 11.9 years of age and diagnosed with T2DM excluded from this study.

Fifty-seven (57.6%) subjects in Group-A, and 39 (46.4%) in Group-B were males. Mean age (years) of subjects was 12.7±5.2 (range 3-23) in Group-A, and 18.7±9.0 (range 5-69) in Group-B. Mean duration of diabetes (years) was 0.6±0.6 (range 0-1) and 7.7±5.5 (range 1-23) in Group-A and B, respectively. Family history of T1DM and T2DM was 1(1%) and 27(27.3%) in Group-A, and 8(9.5%) and 21(25.0%) in Group-B, respectively. Twenty-one (21.2%) subjects presented with DKA in Group-A and 18 (21.4%) in Group-B. Mean±SD for HbA1c was non-significantly higher in Group-A 11.12±2.31 compared to Group-B 10.42±1.45. Mean±SD for C-peptide was 1.91±1.53 ng/mL (0.60±0.48 nmol/L) in Group-A and 1.82±1.01ng/mL (0.57±0.32nmol/L) in Group-B with p=0.984 (Table-I).

**Table-I:** Baseline and clinical parameters of subjects with duration of T1DM.

Parameters	Group-A	Group-B	P-value	Overall
n	99	84	-	183
Age(years)	12.3±5.2	18.7±9.0	<0.0001	15.5±7.8
***Gender***				
Female	42(42.4%)	45(53.6%)	0.132	87(47.5%)
Male	57(57.6%)	39(46.4%)		96(52.5%)
BMI (kg/m2)	16.51±3.3	21.57±4.3	<0.0001	18.88±4.57
Duration of DM (years)	0.64±0.6	7.65±5.5	<0.0001	3.83±5.1
***Family history of T1DM***				
No	98(99%)	76(90.48%)	0.02	174(95.1%)
Yes	1(1%)	8(9.52%)	9(4.9%)
***Family history of T2DM***				
No	72(72.7%)	63(75%)	0.857	135(73.8%)
Yes	27(27.3%)	21(25%)		48(26.2%)
HbA1c (%)	11.12±2.31	10.42±1.45	0.756	10.77±1.88
C-peptide (ng/dl)	1.91±1.53	1.82±1.01	0.984	1.87±1.32
***Diabetic Ketoacidosis (DKA)***				
No	78(78.8%)	66(78.6)	0.884	144(78.7%)
Yes	21(21.2%)	18(21.4%)		39(21.3%)

Data presents as mean ± SD or n (%), P<0.05 considered to be statistically significant.

In Group-A and B, two (2%) and one (1.2%) subjects had a C-peptide value <0.4 ng/mL (<0.13 nmol/L), 10(10%) and 9(10.7%) between 0.4-0.8 ng/mL (0.13-0.26 nmol/L), and 87(87.9%) and 74(88.1%) had >0.8 ng/mL (>0.26 nmol/L), respectively (Table-II).

**Table-II:** Frequency of C-peptides in subjects with duration of T1DM.

C-peptide	Group-A	Group-B	P-value	Overall
n	99	84	-	183
<0.4	2 (2%)	1 (1.2%)	0.902	3 (1.6%)
0.4-0.8	10 (10.1%)	9 (10.7%)		19 (10.4%)
>0.8	87 (87.9%)	74 (88.1%)		161 (88%)

Data presents as n (%), P<0.05 considered to be statistically significant.

**Fig.1:**
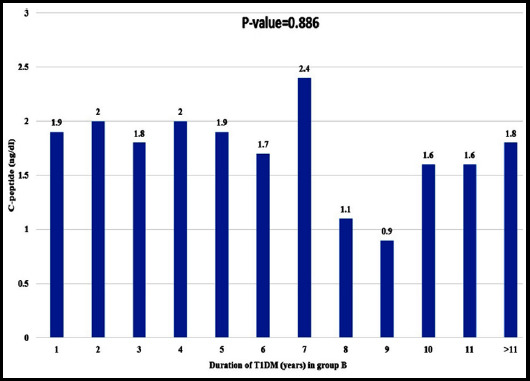
Changes of C-peptide with duration of T1DM in Group-B.

The relationship of C-peptide with duration of diabetes in Group-B was shown in Fig-1. C-peptides in Group-B were seen in 1-year, 2-years, 3-years, 4-years, 5-years, 6-years, 7-years, 8-years, 9-years, 10-years, 11-years and >11-years duration of diabetes as 1.9 ng/mL (n=3), 2.0 ng/mL (n=6), 1.8 ng/mL (n=10), 2.0 ng/mL (n=9), 1.9 ng/mL(n=9), 1.7 ng/mL (n=10), 2.4 ng/mL (n=5), 1.1 ng/mL (n=5), 0.9 ng/mL (n=1), 1.6 ng/mL(n=4), 1.6 ng/mL (n=3), and 1.8 ng/mL (n=19), respectively.

## DISCUSSION

Generally, in T1DM, C-peptide secretion declines with time and the rate of decline is steeper in younger age of onset (less than 10 years), subsequently C-peptide decline at very fast pace.^15^ Data of this study suggests that maintenance of C-peptide levels is much more common in young people with T1DM in Pakistan. Our study found that C-peptide levels at diagnosis were generally higher than those seen in studies in Caucasian populations and did not suggest a clear decline with time.^16^ Similarly, Tanaka S et al., found that the measurement of serum C-peptide values at onset is highly effective for diagnosis of fulminant TIDM- a novel type of T1DM without measuring pancreatic autoantibodies.^17^

In Leighton E et al. study, C-peptide has been shown to correlate with type of diabetes, duration of disease, and age at the time of diagnosis.^7^ In this comparative study, C-peptide level was not stabilized, but found within the normal range in patients with long duration of diabetes (Group-B). These results are consistent with those from the Shields BM et al. study that observed stabilization of C-peptide levels at around seven years after diagnosis and suggest there are important and previously unrecognized changes in immune function and/or beta cell viability around this time that may have critical implications for future pharmaceutical interventions.^18^ Hao et al., found longer duration of follow-up is likely to provide stronger evidence of the effect of disease modifying therapy on preservation of β-cell function of such individuals especially, children have significant insulin secretion after 4 years, but <5% of all individuals of any age maintain their baseline C-peptide levels.^19^

The male excess seen in this study in Group-A (a consecutive series) is consistent with the findings by Shera et al.^20^ DKA was non-significantly observed at presentation in Group-A and B, similar to Razavi et al., study.^21^ Patients have strong family history of T2DM in our study as compared to family history of T1DM, which reflect current burden of T2DM as 26.3% reported by Basit et al.^22^ The recent epidemic of T2DM in South Asian region is thought to be largely due to environmental factors such as unhealthy diets, increasing body weight and physical inactivity coupled with a strong genetic predisposition. Moreover, babies are often born smaller because of under nutrition, which when coupled with subsequent obesity, further rises risk of insulin resistance in their later life.^23^

Certain features support a diagnosis of T2DM, for instance; the hallmark of T2DM is obesity with up to 85% of affected children at diagnosis are either overweight or obese. Sometimes, obesity may be masked by substantial weight loss in the months or years preceding diagnosis.^24^ In contrast, the hallmark of T1DM is the selective damage of beta cells in the pancreas by autoimmunity, or insulitis.^19^ However, marked heterogeneity exist in the clinical features of both T1DM and T2DM. Subjects with non-immune-mediated T1DM may have clinical features indistinguishable from immune-mediated T1DM. Proper management of diabetes depends on its type and therefore it is very important to classify a patient’s diabetes correctly, as oral hypoglycemic drugs can be used in T2DM. The American Diabetes Association committee recommended the term type 1A for immune mediated diabetes and type 1B for non-immune mediated diabetes.^25^ Higher frequency of type 1B diabetes (45%) due to low frequency of IA-2 antibody was observed by Balasubramanian K et al., among children and adolescents with recent-onset T1DM than previously reported in other Caucasian populations.^26^ There is little information on type 1A and type 1B diabetes in other racial groups. In our study, subjects were not further categorized into type 1A and type 1B which is the limitation of our study.

### Further limitations of the study are

1. The diagnosis was based on clinical features. Due to financial constraints the tests of patients’ T1DM autoantibodies and insulin levels to label TIDM biochemically and immunologically were not performed in Group-B. The results of these tests in Group-A have been separately published.^11^
2. Other antibody assays to support autoimmunity as a cause were not done.
3. Regional normality of C-peptide levels were not known.

Despite having certain limitations, this is perehaps the first research on this topic conducted in Pakistan. As mentioned in methodology that it was an additional investigation from the main study has been published earlier that focused only the subjects with <1-year duration of T1DM. Here, in this study, we compare the two groups subjects with < 1-year duration of T1DM and subjects with >1-year duration of T1DM. Hence, comparing available data between two groups in this part of the world may help researchers for future planning.

## CONCLUSION

The study found that subjects with longer duration of T1DM had non-significantly decreased C-peptide levels compared to a group in which C-peptide was measured at or soon after diagnosis. Furthermore, C-peptide levels in many subjects with longer duration were higher than expected in classic T1DM. Longitudinal studies with various ethinic Group-Are needed to more fully understand the pathophysiology in these cases. Such studies would include measurement of autoantibodies and repeated measurement of C-peptide.

### Author’s Contribution

**AF:** Concept, design, literature search, approval of final manuscript and responsible for accuracy or integrity of work.

**NW:** Literature search, interpretation of data, and wrote the manuscript.

**SA:** Literature search, edited and approval of the final manuscript.

**GDO:** Concept, design, edited and approval of the final manuscript

**YA:** Concept, design, edited and approval of the final manuscript and responsible for accuracy or integrity of work.

**AB:** Concept, design, edited and approval of the final manuscript.
